# Microbiome-derived secondary bile acids promote repair of colonic mucosa after injury

**DOI:** 10.1038/s44321-025-00218-2

**Published:** 2025-03-21

**Authors:** Brecht Attema, Folkert Kuipers

**Affiliations:** European Research Institute for the Biology of Ageing (ERIBA), University of Groningen, University Medical Center Groningen, Groningen, The Netherlands

**Keywords:** Digestive System, Metabolism, Microbiology, Virology & Host Pathogen Interaction

## Abstract

B. Attema and F. Kuipers discuss a recent study by Schoonjans and colleagues(in this issue of EMBO Mol Med) that reveals the important role of secondary bile acid signaling in colon mucosal healing after injury.

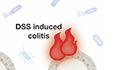

## Teaser

Dysbiosis, i.e., an imbalance in the composition of the intestinal microbiome, has been linked to the etiology of inflammatory bowel diseases (IBD). Secondary bile acids (BAs) are microbiome-derived metabolites increasingly recognized for their important signaling functions in host (patho)physiology. In this issue of *EMBO Molecular Medicine*, Jalil et al (Jalil et al, 2025) report that increased production of secondary BAs upon administration of the human bacterium *Clostridium scindens* (*C. scindens*) improves colonic health in mice with chemically-induced colitis via activation of the membrane bound BA receptor TGR5. These findings indicate an important role for secondary BA signaling in colon mucosal healing upon injury and suggest that modulation of microbiome composition could serve as a therapeutic approach for patients with IBD.

Primary BAs, i.e., cholic acid (CA) and chenodeoxycholic acid (CDCA), are synthesized in the liver, secreted into the bile and, upon ingestion of a meal, released into the intestine to facilitate absorption of lipids by their detergent actions. While the majority of primary BAs are subsequently reabsorbed in the ileum, some reach the colon, where their structures can be modified by bacteria. Certain bacterial species can modify primary BAs through dehydroxylation, epimerization, or oxidation reactions, resulting in the formation of secondary BAs, including deoxycholic acid (DCA) and lithocholic acid (LCA). These secondary BAs can be absorbed from the colon and re-enter the circulating BA pool. In addition to acting as intestinal soaps, BAs serve as important signaling molecules in the regulation of lipid and glucose metabolism and inflammation. These effects are mediated through the activation of nuclear receptors, such as farnesoid X receptor (FXR) and vitamin D receptor (VDR), and the membrane-bound Takeda G protein-coupled receptor 5 (TGR5).

Patients with ulcerative colitis (UC), a subtype of IBD, have been shown to have lower fecal levels of secondary BAs compared to healthy controls (Sinha et al, 2020), but their role in disease development or progression remains unclear. In the current issue, Jalil et al evaluated the impact of the administration of *C. scindens*, a 7α-dehydroxylating bacterium converting CA and CDCA into DCA and LCA, respectively, on BA composition and disease severity in a mouse model of dextran sulfate sodium (DSS)-induced colitis (Jalil et al, 2025). Two distinct mouse models were used: the Oligo-MM^12^ gnotobiotic mouse model devoid of 7α-dehydroxylating bacteria and vancomycin-treated specific pathogen-free (SPF) mice. In both models, administration of *C. scindens* resulted in higher levels of secondary BAs in plasma and in feces, without altering the BA pool or hydrophobicity index. *C. scindens* administration during DSS-induced colitis resulted in both models in faster weight recovery and promotion of intestinal regeneration. Furthermore, in vancomycin-treated SPF mice, *C. scindens* administration resulted in reduced disease severity and improved intestinal permeability, highlighting the potential therapeutic value of *C. scindens* administration in mucosal repair.

TGR5 is ubiquitously expressed in epithelial cells across the entire gastrointestinal tract. Previous work from the same group suggests that TGR5 is also expressed in intestinal stem cells, and as such, might play a role in epithelial regeneration (Sorrentino et al, 2020). Therefore, the authors utilized whole-body TGR5-knockout mice and demonstrated that the protective effects of *C. scindens* administration were abolished in the absence of TGR5. RNA sequencing revealed induction of pathways related to intestinal stem cell proliferation in colons of *C. scindens*-treated mice with intact TGR5. In contrast, these pathways were downregulated in TGR5-knockout mice, indicating that, indeed, TGR5 is critical for intestinal regeneration upon injury by inducing stem cell proliferation. Computational analyses of public datasets from UC patients revealed that *Tgr5* expression was not altered in the colon and rectum compared to healthy subjects, while expression of inflammatory pathways were increased. Positive correlations were found between the levels of fecal unconjugated 7α-dehydroxylated BAs (DCA and LCA) and intestinal epithelial cell specification in UC patients.

**Figure 1:**
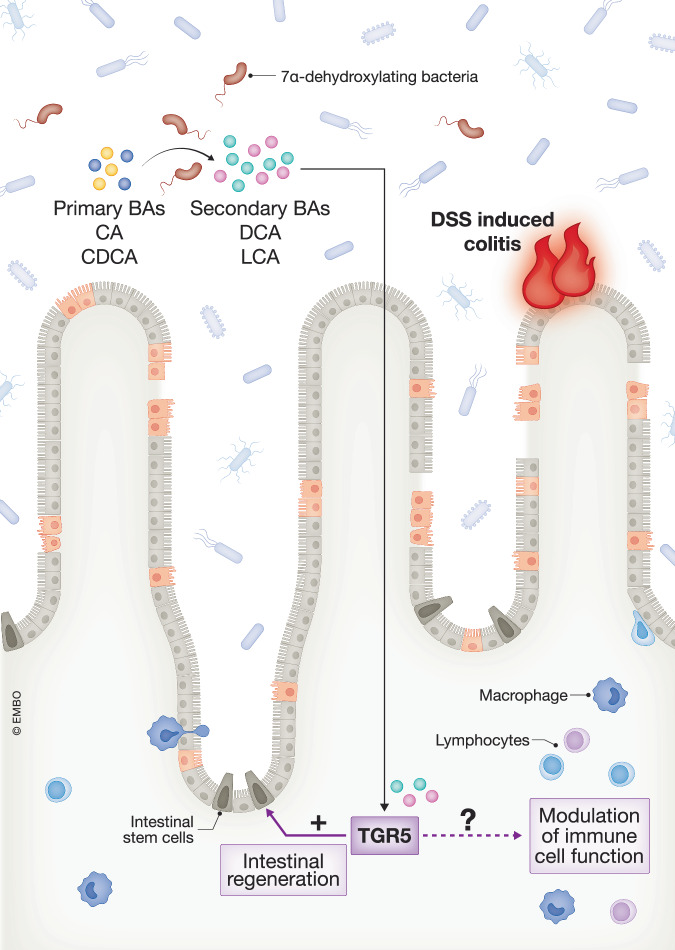
Secondary BAs can promote intestinal regeneration upon colonic injury via TGR5 in mice. Primary BAs (CA, CDCA) are synthesized in the liver and released into the intestine, where they interact with the gut microbiome. Specific 7α-dehydroxylating bacteria, such as Clostridium scindens, can structurally convert primary BAs into secondary BAs (mainly DCA and LCA). These secondary BAs can be absorbed in the colon and activate the TGR5 receptor. During DSS-induced colitis in mice, elevated levels of secondary BAs can promote mucosal repair and intestinal regeneration via TGR5 signaling, alleviating disease severity. Next to the promotion of intestinal regeneration, secondary BAs may also contribute to mucosal healing by dampening inflammation through TGR5-mediated modulation of immune cell function.

The protective role of secondary BAs in mucosal repair has been demonstrated by others (Liu et al, 2022). Beyond promoting intestinal regeneration, it has been shown that secondary BAs can modulate immunity and colonic inflammation (Kuipers et al, 2020). Indeed, several BA-responsive receptors, including TGR5, FXR, and VDR are known to be expressed in immune cells. Various secondary BA isoforms are thought to dampen inflammation through diverse mechanisms, such as promoting the differentiation of colonic lamina propria-resident Foxp3^+^ regulatory T (Treg) cells (Hang et al, 2019; Li et al, 2021), and inhibiting the differentiation of proinflammatory T helper 17 (Th17) cells (Hang et al, 2019). Combined with the current work (Jalil et al, 2025), these data highlight the protective role of secondary BAs in modulating colitis severity, potentially mediated by both enhanced intestinal regeneration and immune cell modulation. Since the current study employed a whole-body TGR5-knockout model, future studies aimed at identifying the specific cell type primarily responsible for the TGR5-mediated effects of LCA could further elucidate the exact mechanism underlying improved colonic regeneration (Fig. 1). It is important to note that differences in BA metabolism between mice and humans may hamper direct translation to colitis patients. The high abundance of hydrophilic muricholic acids (MCAs) in the murine BA pool and effective rehydroxylation of secondary BAs in murine liver by Cyp2c70 and Cyp2a12, respectively, strongly impacts on overall signaling properties of the circulating BA pool (Honda et al, 2020; de Boer et al, 2020).

Interestingly, the capacity of (secondary) BAs to promote regenerative capacity in the intestine, as shown in this issue, aligns with the emerging notion that secondary BAs may contribute to healthy longevity. Recent work by Qu and colleagues provides evidence for the potential anti-aging effects of LCA, showing an extension of lifespan in Drosophila, *C. elegans*, and mice, likely via activation of AMP-activated protein kinase (AMPK), thereby mimicking the longevity effect of caloric restriction (Qu et al, 2024). Intriguingly, the human BA pool shows great intraindividual variations in size and composition, partly influenced by the genetic make-up of the gut microbiome (Wang et al, 2021) making it amendable for interventions. Hence, it is expected that expanding knowledge in the field of *Bile-ology* will contribute to the design of new intestinal health-promoting strategies in the years to come.
